# Physical activity and quality of life in long-term hospitalized patients with severe mental illness: a cross-sectional study

**DOI:** 10.1186/s12888-017-1466-0

**Published:** 2017-08-18

**Authors:** Jeroen Deenik, Frank Kruisdijk, Diederik Tenback, Annemarie Braakman-Jansen, Erik Taal, Marijke Hopman-Rock, Aartjan Beekman, Erwin Tak, Ingrid Hendriksen, Peter van Harten

**Affiliations:** 1 GGz Centraal, Utrechtseweg 266, 3818 EW Amersfoort, The Netherlands; 2 Faculty of Behavioural, Management and Social sciences, University of Twente, Drienerlolaan 5, 7522 NB Enschede, The Netherlands; 3 School for Mental Health and Neuroscience Maastricht University, Minderbroedersberg 4-6, 6211 LK Maastricht, The Netherlands; 4 Body@Work, TNO-VU University Medical Centre, Van der Boechorststraat 7, 1081 BT Amsterdam, The Netherlands; 5 Department of Pharmacoepidemiology and Clinical Pharmacology, Utrecht University, Universiteitsweg 99, 3584 CG Utrecht, The Netherlands; 6 The Netherlands Organization for applied scientific research TNO, Schipholweg 77-89, 2316 ZL Leiden, The Netherlands; 7 EMGO institute for Health and Care Research, Department of Public and Occupational Health, VU University Medical Centre, Van der Boechorststraat 7, 1081 BT Amsterdam, The Netherlands; 8 Department of Psychiatry, VU University Medical Centre, A.J. Ernststraat 1187, 1081 HL Amsterdam, The Netherlands

**Keywords:** Schizophrenia, Physical activity, Accelerometry, Inpatients, Quality of life, Attitude; self-efficacy

## Abstract

**Background:**

Increasing physical activity in patients with severe mental illness is believed to have positive effects on physical health, psychiatric symptoms and as well quality of life. Till now, little is known about the relationship between physical activity and quality of life in long-term hospitalized patients with severe mental illness and knowledge of the determinants of behavioural change is lacking. The purpose of this study was to elucidate the relationship between objectively measured physical activity and quality of life, and explore modifiable psychological determinants of change in physical activity in long-term hospitalized patients with severe mental illness.

**Methods:**

In 184 inpatients, physical activity was measured using an accelerometer (ActiGraph GTX+). Quality of life was assessed by EuroQol-5D and WHOQol-Bref. Attitude and perceived self-efficacy towards physical activity were collected using the Physical Activity Enjoyment Scale and the Multidimensional Self Efficacy Questionnaire, respectively. Patient and disease characteristics were derived retrospectively from electronic patient records. Associations and potential predictors were analysed using hierarchical regression.

**Results:**

Physical activity was positively related with and a predictor of all quality of life outcomes except on the environmental domain, independent of patient and disease characteristics. However, non-linear relationships showed that most improvement in quality of life lies in the change from sedentary to light activity. Attitude and self-efficacy were not related to physical activity.

**Conclusions:**

Physical activity is positively associated with quality of life, especially for patients in the lower spectrum of physical activity. An association between attitude and self-efficacy and physical activity was absent. Therefore, results suggest the need of alternative, more integrated and (peer-)supported interventions to structurally improve physical activity in this inpatient population. Slight changes from sedentary behaviour to physical activity may be enough to improve quality of life.

## Background

Research into lifestyle-related factors in patients with Severe Mental Illness (SMI) is increasing. Efforts often focus on physical health, consistent with the highly prevalent metabolic risk factors [1] that underlie the well-known poor cardiovascular health and premature mortality gap of at least 10–20 years compared to the general population [2, 3]. Recent systematic reviews and meta-analyses showed beneficial effects of increasing physical activity (PA) on physical health, psychiatric symptoms, global functioning, and quality of life (QoL) [4–7]. These are relevant findings, especially for long-term hospitalized patients with SMI, given the combination of a strong negative relationship between illness duration and QoL [8] and a high level of sedentariness in this population [9].

Although QoL is a complex construct influenced by multiple factors [10], these recent findings positively support PA as an important modifiable factor affecting patients’ QoL.

However, until now little is known about the relationship between objective measured PA and QoL in long-term hospitalized patients with SMI and most of the studies in the referred systematic reviews and meta-analyses involved outpatient- or short term hospitalized populations. Objective PA measurement of patients with long-term hospitalization due to their illness severity remains a challenge and just a few studies succeeded in such measurements at larger scale [9, 11, 12].

Also, despite growing awareness of the positive effects of increasing PA in patients with SMI, no clear answer exists on how to motivate patients with SMI to structurally engage in PA [13]. In addition, the hospital setting has been considered ‘obesogenic’ and a cause of inactivity itself [14, 15]. A long term stay therefore negatively affects PA due to regulated inactivity. More knowledge of determinants of change in PA in this population is needed. The Theory of Planned Behaviour [16], a basic social-cognitive model, provides insight into such determinants. It distinguishes attitudes towards the desired behaviour and its outcomes, subjective norms (perceived social pressure and (dis)approval of certain behaviours) and perceived behavioural control [16], also defined as self-efficacy [17]. These determinants are assumed to affect one’s intention to perform the given behaviour, which is hypothesized to be the immediate antecedent of the actual behaviour (e.g. being more physically active). Especially in long-term mental healthcare, it is important to gain more insight into psychiatric and psychological determinants of PA to guide activation [18]. However, in SMI patients the abovementioned subjective norms and the ability to consciously establish intentions towards behaviour are affected by a lack of insight into their condition, poor skills in cognitive empathy and planning deficits [19–21]. Therefore, we decided to explore attitude and self-efficacy as determinants with the greatest potential to influence the result of treatment. To our knowledge, these determinants are rarely studied in a SMI population and have never been associated with objectively measured PA.

The aim of our study was to (A) analyse the relationship between objectively measured PA and QoL in long-term hospitalized patients with severe mental illness and (B) explore to what extent attitude and self-efficacy are related to the level of PA in this population.

## Methods

### Participants

Subjects were long-term hospitalized patients with SMI of a psychiatric hospital in The Netherlands for whom we provide mental healthcare and residence if they are unable to live independently or in sheltered homes. Inclusion criteria were age ≥ 18, hospitalized ≥1 year and treatment history ≥2 years. For the current study, we used accelerometer data previously reported by Kruisdijk et al. [9]. In this relevant study, forty-four patients were excluded because of no informed consent (*n* = 26), unstable psychiatric or physical conditions (*n* = 17) or relocation (*n* = 1). Of the 207 included patients, 23 dropped out because of a lack of required wear time (*n* = 19), losing their accelerometer (*n* = 2), discharge during the study period (*n* = 1) or an acute episode of illness (*n* = 1). In total 184 patients were included with sufficient accelerometer data, that we used in analysis in the current study, which was approved by the Central Committee on Research Involving Human Subjects (CCMO). After extensive verbal explanation per ward, taking understandable comments due to the mental illness into account (e.g. fear, suspicion and psychotic thoughts), written informed consent was obtained from all subjects who understood the intent of the study and were willing to participate.

### Procedures

To measure PA, subjects wore an accelerometer (ActiGraph GT3X+) for five consecutive days (Wednesday morning to Sunday evening) except while sleeping or during water related activities [9]. Questionnaires for QoL and attitude and self-efficacy as determinants of change in PA were added to the routine health screening and conducted by a trained research assistant in a semi-structured interview. The average duration of a screening was 20–30 min, depending on the specific patient. If needed, the screening was split up into two appointments (e.g. due to a limited attention span).

### Measurements

#### Patient- and disease-specific characteristics

Gender, age, years of hospitalization, diagnosis, illness severity and use of medication were derived retrospectively from electronic patient records. Primary diagnoses were classified following the DSM-IV-TR in main groups of the most frequent diagnoses: schizophrenia and other psychotic disorders, personality disorders and affective disorders. Less frequent diagnoses were merged in ‘others’. Furthermore, affective disorders were split into depression and bipolar disorder. Use of antipsychotics was split into first, second or both generations, because the difference in side-effects (movement disorders and metabolic side-effects, respectively) may have an effect on the activity level. Severity of illness was measured by the Dutch translation of the severity-scale of the Clinical Global Impression Scale (CGI, [22]), rating from 1 (normal, not at all ill) to 7 (among the most extremely ill patients).

#### Physical activity

The ActiGraph GT3X+ (*ActiGraph*, Pensacola, Florida, VS) was used to measure PA. The accelerometers were worn on the right hip with an elastic strap between two belt loops. Patients without belt loops used a pouch pinned at the same place. A wear time of ≥6 h/day for ≥3 days was used as the criterion for sufficient measurement. To be able to compare individual data, the same timeframe was used for each dataset: 09.00 am till 10.00 pm. Data were analysed using the ActiGraph software ActiLife 6.8.0 and calculated into average total activity counts per hour (TAC/h) as a continuous and detailed outcome variable of PA, where more counts indicate a higher level of PA. For detailed information on used settings and criteria, see Kruisdijk et al. [9]. The GT3X+ has a high inter- and intra-instrumental reliability and validity [23–25].

#### Quality of life

The Dutch versions of the EuroQol 5D (EQ-5D) and brief World Health Organization Quality of Life Assessment scale (WHOQoL-Bref) were used to assess QoL. As a generic instrument, the EQ-5D measures five dimensions of health (one item each): mobility, self-care, usual activities, pain/discomfort and anxiety/depression, rated from 1 (no problems) to 3 (many problems). The added value of the EQ-5D is the calculation of an index score ranging from 0 (worst QoL) to 1 (perfect QoL) using the Dutch value-set based on time-trade-off weightings of a representative Dutch sample [26]. The EQ-5D is a valid instrument to measure QoL in patients with a diagnosis of schizophrenia [27].

Because the EQ-5D is recommended to be used complementary to other instruments [28] and physical disability and poor daily functioning not necessarily mean that the particular patient has a poor QoL [29], we added the WHOQoL-Bref. This questionnaire focuses on ones’ perceived QoL, including 24 items divided in four domains: physical (7 items), psychological (6 items), social (3 items) and environmental (8 items). In addition, there are two items that individually score one’s overall perception of QoL and satisfaction with his/her health. Items are scored on a five-point Likert scale (1 to 5; e.g. from ‘very dissatisfied’ to ‘very satisfied’). Scores were transformed into domain scores ranging from 4 to 20, according to the WHO guidelines. The WHOQoL-Bref is the most often used QoL-instrument in studies investigating patients with a diagnosis of schizophrenia [30] and showed good to excellent reliability and validity in these patients in long-term mental healthcare [31] and in Dutch outpatients with psychiatric disorders [32]. In the present study, we found a sufficient internal consistency in the separate domains: physical (α = 0.74), psychological (α = 0.82), social (α = 0.63) and environmental (α = 0.72).

#### Attitude & self-efficacy towards physical activity

For these measures, no validated Dutch questionnaires were available for this population. Therefore, attitude was measured using the Dutch version of the Physical Activity Enjoyment Scale (PACES; [33]), previously used in the general population [34]. It includes 18 dichotomous statements regarding the participants’ thoughts about physical activities they do (or used to do). Items are scored at a Likert-scale from 1 to 7 whereby participants choose the number that most closely corresponds to the way they feel (e.g. ‘I feel bored’ vs. ‘I feel interested’). Eleven items are formulated negatively. After recoding these items, a sum-score was calculated, with a higher score representing a more positive attitude towards PA (range 18–126). In the present study, the questionnaire showed a high internal consistency (α = .97).

Self-efficacy was measured with a questionnaire based on the Multidimensional Self Efficacy Questionnaire (MSEQ; [35]). Originally, this questionnaire comprised 18 items measuring the extent to which a patient feels able to be physically active (e.g. ‘I am confident in my ability to exercise when I am under a lot of stress’). Items are scored on a Likert-scale from 1 (totally unable) to 5 (totally able). For use in the present study, the questionnaire was translated (once forwards) into Dutch. To avoid unduly burdening patients with irrelevant questions and to have a questionnaire in line with the treatment conditions, some adjustments were made. First, using understandable terminology towards patients, we replaced the word ‘exercise’ by ‘be physically active’. Secondly, due to the inpatient setting, the item regarding PA when traveling became unnecessary and was deleted, and items referring to several social situations were reduced to one item focusing on the situation at the treatment group (I am confident in my ability to be in PA when my fellow group members don’t want me to go to physical activities). Finally, one of the three items regarding cold weather conditions was adjusted into an item on hot weather, in order to involve summer conditions. A sum score was used with a higher score representing a higher self-efficacy towards PA (range 15–75). In the present study, the adjusted questionnaire showed a high internal consistency (α = 0.92).

### Statistical analysis

Data analyses were performed using SPSS 22.0. Continuous variables were examined for normality and homogeneity by comparing means with medians and standard deviations, and by analysing frequency histograms and normality plots. Linearity was examined by scatterplots and analysing the variables in quartiles. If variables were not distributed linearly with respect to the dependent variables, they were added as quartiles in the analysis, with the first quartile as reference-category. Categorical variables (diagnosis and types of antipsychotics) were transformed into dummy variables. ‘Schizophrenia and other psychotic disorders’ and ‘first generation antipsychotics’ were the reference groups for diagnosis and antipsychotics, respectively.

Relationships between the independent (PA, attitude or self-efficacy) and dependent variable (QoL outcomes or PA) were analysed using linear regression. First, analyses focused on these associations specifically. To estimate these associations as accurately as possible, it was checked whether patient and disease characteristics affected the relationship. This confounding was defined as a change of ≥10% in the regression coefficient of the particular independent variable (e.g. PA) when adding a patient or disease characteristic. The strongest confounder was added to the model to control for, until there were no variables left causing ≥10% change in the regression coefficient. For the analysis of PA, we also controlled for the way of attachment of the accelerometer. Associations were considered significant at a 0.05 two tailed significance level. Then, regardless of any specific association, significant predictors of QoL and PA were analysed using multiple regression with manual backward elimination retaining only the strongest predictors (*p* < 0.10). Proportions of explained variance were reported to give an indication of the predictive power of these models. Multicollinearity was examined by observing regression coefficients (e.g. change of direction when adding variables) and collinearity statistics (VIF > 10 and tolerance <0.2). Because PA is represented in large numbers by total activity counts per hour, the variable was standardized (each value subtracted by the mean total activity counts per hour and divided by its SD) to use as independent variable in analysis towards QoL, in order to optimize readability.

## Results

Table 1 shows the characteristics of the 184 participants. They were between 25 and 91 years old and hospitalized with a minimum of one and a maximum of 58 years.

**Table 1:** Patient and disease characteristics, physical activity, quality of life, attitude and self-efficacy (*N* = 184)

Variable (scale)	Mean (SD)		N (%)	
Gender (men)			108	(58.7)
Age (years)	57.4	(12.8)		
Diagnosis				
Schizophrenia and other psychotic disorders			142	(77.2)
Personality disorder			17	(9.2)
Mood disorder				
Depressive			6	(3.3)
Bipolar			7	(3.8)
Others^a^			12	(6.5)
Years of hospitalization, median (25–75 percentile)^b^	6.0	(4.0–21.8)		
Severity of illness (range 1–6)	4.7	(1.4)		
Medication				
Antipsychotics				
First generation			36	(19.6)
Second generation			70	(38.0)
Both			73	(39.7)
Antidepressants			82	(44.6)
TAC/h	24,527	(14821)		
EQ-5D index score (0–1)	0.6	(0.3)		
WHOQoL-Bref				
Overall QoL rating (1–5)	3.5	(0.9)		
Health satisfaction rating (1–5)	3.4	(1.1)		
Physical domain (4–20)	13.6	(2.7)		
Psychological domain (4–20)	13.1	(3.1)		
Social domain (4–20)	13.1	(2.9)		
Environmental domain (4–20)	14.9	(2.1)		
Attitude towards PA (18–126)	76.6	(26.8)		
Self-efficacy towards PA (15–75)	41.2	(14.6)		

*TAC/h* average Total Activity Counts per hour, *QoL* Quality of life, *EQ-5D* EuroQol five-dimension questionnaire, *WHOQoL-Bref* World Health Organization Quality of Life questionnaire, brief version

^a^Delirium, dementia, and amnestic and other cognitive disorders (*n* = 3); substance-related disorders (*n* = 3), somatoform disorders (*n* = 2); mental disorder not otherwise specified (*n* = 2); anxiety disorder (*n* = 1); developmental disorder (*n* = 1)

^b^Positively skewed distribution

### Physical activity and quality of life

**Fig. 1:**
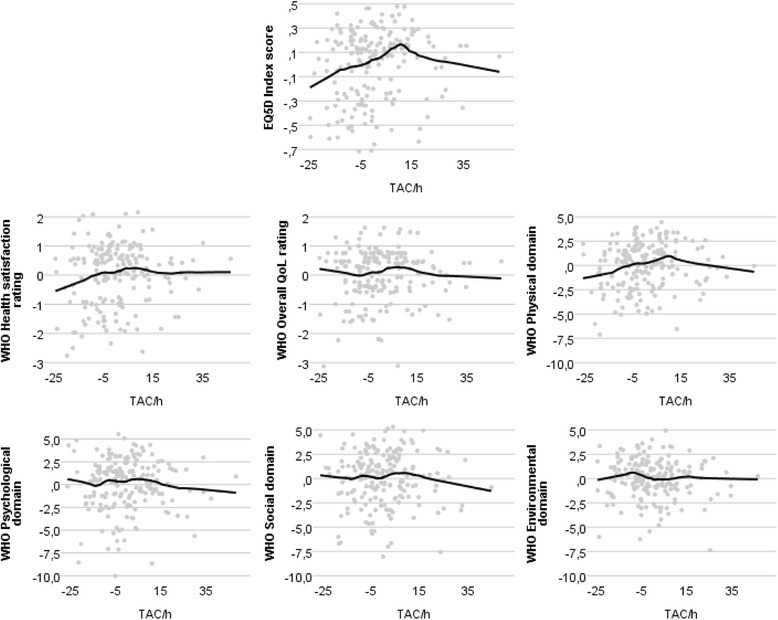
Non-linear distributions between physical activity (TAC/h) and Quality of Life (QoL) variables. Explored using scatterplots and LOESS Curves (Kernel: Epanechnikov, = 0.5) on standardized axis. TAC/h ×1000. TAC/h = average Total Activity Counts per hour; QoL = Quality of Life; LOESS = Locally Estimated Scatterplot Smoothing; EQ5D = EuroQol-5D; WHO = World Health Organisation

When examining scatterplots, we found that PA was non-linearly distributed towards QoL. Although there was no obvious alternative shape of the distribution (e.g. quadratic), smoothing a line using Locally Estimated Scatterplot Smoothing (LOESS) [36] clearly visualized that QoL did not constantly increase with more PA (Fig. 1). Coefficients of PA quartiles confirmed this finding (Table 3). Because of this non-linearity, quartiles were used in the regression analysis, with the lowest quartile (Q1) as reference group. To support interpretation of the results, Table 2 provides insight into means of activity counts and corresponding intensities within the quartiles, according to age-specific cut-off points for tri-axial measurement [37–39].

**Table 2:** Average total activity counts per hour and proportions of wear time in intensities within quartiles

Quartile	TAC/h				Intensities^c^		
	Mean (SD)		min^b^	max^b^	% SB	% LPA	% MVPA
Q1^a^	8260	(2938)	2518	12,840	92.2	6.2	1.6
Q2	18,136	(2973)	13,350	22,317	86.0	10.1	3.9
Q3	27,160	(2663)	22,566	31,469	81.9	11.7	6.4
Q4	44,553	(11814)	31,702	83,698	74.4	14.0	11.6

*Q* Quartile (*n* = 46), *TAC/h* average Total Activity Counts per hour, *SB* Sedentary Behaviour, *LPA* Light Physical Activity, *MVPA* Moderate to Vigorous Physical Activity

^a^reference group in analysis

^b^minimum and maximum average TAC/h (patient) within the quartile

^c^mean percentage of wear time that patients within the quartile spend in: SB [<150], LPA [151–3207], MVPA [≥3208; but ≥2751 for 65 years or older] [37–39]. Thresholds in counts per minute

Table 3 shows the associations between PA (total activity counts per hour) and QoL with the corresponding confounders, reflected in regression coefficients for the particular associations and *F*-values for the models as a whole. There were significant positive associations between PA and the EQ-5D index score and WHO overall, physical, psychological and social QoL. For overall QoL and the psychological and social domains, patients with PA levels in the lower spectrum (Q2) had the best QoL and the QoL of the most active patients (Q4) differed less from the least active patients (Q1, reference group). This corresponds to the shapes of distribution in the uncorrected data, showing a (small) increase which then runs off. There were no significant associations with health satisfaction and environmental QoL.

**Table 3:** Associations between outcomes, uncorrected and corrected for confounding patient and disease characteristics

	Corrected for							Uncorrected			Corrected		
Variable	A	B	C	D	E	F	G	B	(95% CI)	*F*	B	(95% CI)	*F*
Associations between physical activity (TAC/h) and QoL													
EQ-5D index score		B		D						3.89*			4.81***
Q2								0.16*	(0.03–0.28)		0.18**	(0.06–0.31)	
Q3								0.14*	(0.01–0.26)		0.18**	(0.05–0.31)	
Q4								0.20**	(0.08–0.33)		0.19**	(0.05–0.33)	
WHOQoL-Bref													
Overall QoL rating		B		D						2.78*			3.20**
Q2								0.54**	(0.17–0.92)		0.60**	(0.21–0.98)	
Q3								0.30	(−0.07–0.68)		0.40*	(<0.01–0.81)	
Q4								0.33	(−0.05–0.70)		0.35	(−0.08–0.78)	
Health satisfaction rating		B	C	D						1.04			2.62**
Q2								0.39	(−0.07–0.85)		0.47	(−0.01–0.94)	
Q3								0.26	(−0.20–0.72)		0.44	(−0.05–0.93)	
Q4								0.30	(−0.16–0.77)		0.50	(−0.03–1.02)	
Physical domain	A	B	C	D						2.75*			3.72***
Q2								1.06	(−0.02–2.13)		1.18*	(0.10–2.26)	
Q3								1.18*	(0.10–2.26)		1.66**	(0.54–2.79)	
Q4								1.47**	(0.39–2.54)		1.50*	(0.31–2.69)	
Psychological domain	A	B		D			G			0.90			5.22***
Q2								0.88	(−0.39–2.15)		1.35*	(0.11–2.59)	
Q3								0.45	(−0.82–1.72)		1.01	(−0.29–2.31)	
Q4								−0.03	(−1.30–1.24)		0.45	(−0.93–1.83)	
Social domain		B	C							2.06			2.06*
Q2								1.42*	(0.23–2.62)		1.46*	(0.19–2.73)	
Q3								1.13	(−0.07–2.33)		1.38*	(0.07–2.69)	
Q4								0.75	(−0.44–1.95)		1.27	(−0.12–2.67)	
Environmental domain	A	B	C	D	E	F				0.18			1.37
Q2								0.23	(−0.66–1.12)		0.04	(−0.92–0.99)	
Q3								0.29	(−0.60–1.18)		0.21	(−0.78–1.19)	
Q4								0.08	−0.81 – 0.97)		−0.16	(−1.20–0.89)	
Associations between attitude and self-efficacy and physical activity (TAC/h) ^a^													
Attitude towards PA		B		D				83.60*	(3.58–163.62)	4.25*	36.54	(−34.91–107.99)	14.09***
Self-efficacy towards PA		B	C					82.52	(−64.98–230.01)	1.22	67.39	(−59.69–194.48)	11.24***

Significant results shown in bold. A, gender; B, age; C, diagnosis; D, illness severity; E, years of hospitalization; F, use of antipsychotics; G, use of antidepressants. B = regression coefficient; Q = Quartile (*n* = 46); TAC/h = average Total Activity Counts per hour

*p < 0.05. **p < 0.01. ***p < 0.001

^a^also corrected for the way of accelerometer-attachment

The multiple regression with backward elimination showed PA to be a significant predictor for the EQ-5D, WHO overall QoL, health satisfaction and the physical, psychological and social domains (Table 4). Regression coefficients for the prediction of the WHO overall, psychological and social QoL showed the strongest relationships for the lower spectrum of activity (Q2). This confirmed the distributions we found earlier (Fig. 1). Explained variance (*R* ^*2*^_*Adj*_) of the models of these significant predictors ranged between 8% and 17%. The level of PA was no significant predictor for environmental QoL, which was predicted by years of hospitalization, illness severity and use of antipsychotics (*F* = 3.91, *p* < 0.01, *R* ^*2*^_*Adj*_ = 0.06).

**Table 4:** Multiple regression models with significant predictors of quality of life (*p* < 0.10), using backward-selection

Measure	B	95% CI	*F*	*R* ^*2*^	*R* ^*2*^_*Adj*_ *.*
Predicting EQ-5D index score					
2003Overall model			6.76****	0.16	0.14
TAC/h					
Q2	0.16***	(0.05–0.28)			
Q3	0.15**	(0.04–0.27)			
Q4	0.17***	(0.05–0.28)			
Gender					
Illness severity					
Predicting WHO overall QoL rating					
Overall model			5.18****	0.13	0.10
TAC/h					
Q2	0.56***	(0.20–0.92)			
Q3	0.35*	(−0.01–0.71)			
Q4	0.22	(−0.14–0.59)			
Gender					
Illness severity					
Predicting WHO health satisfaction rating					
Overall model			2.74***	0.19	0.12
TAC/h					
Q2	0.48**	(0.01–0.95)			
Q3	0.41	(−0.08–0.90)			
Q4	0.50*	(−0.02–1.01)			
Age^a^					
Diagnosis					
Illness severity^a^					
Antidepressants					
Predicting WHO physical QoL domain					
Overall model			3.72****	0.24	0.17
TAC/h					
Q2	1.18**	(0.10–2.26)			
Q3	1.66***	(0.54–2.79)			
Q4	1.50**	(0.31–2.69)			
Age^a^					
Gender					
Diagnosis					
Illness severity^a^					
Predicting WHO psychological QoL domain					
Overall model			3.85****	0.23	0.17
TAC/h					
Q2	1.09*	(−0.16–2.35)			
Q3	0.90	(−0.39–2.20)			
Q4	0.30	(−1.07–1.68)			
Age					
Gender					
Diagnosis					
Illness severity					
Use of antipsychotics					
Use of antidepressants					
Predicting WHO social QoL domain					
Overall model			2.33***	0.14	0.08
TAC/h					
Q2	1.49**	(0.27–2.72)			
Q3	1.19*	(−0.03–2.42)			
Q4	0.97	(−0.30–2.23)			
Years of hospitalization					
Diagnosis					
Illness severity^a^					
Use of antidepressants					

Significant results shown in bold. TAC/h = average Total Activity Counts per hour; Q = Quartile (*n* = 46)

*p < 0.10. ** *p* < 0.05. *** *p* < 0.01. *** *p* < 0.001

^a^In quartiles

### Attitude and self-efficacy and physical activity

Table 3 shows the associations between attitude and self-efficacy and PA, reflected in regression coefficients for the particular associations and *F*-values for the models as a whole. After correction, there were no significant associations. In parallel, attitude and self-efficacy were not significant predictors of PA in multiple regression. Of all patient and disease characteristics, only age was a significant predictor (*F* = 38.66, *p* < 0.001, *R* ^*2*^_*Adj*_ = 0.29).

## Discussion

**Fig. 2:**
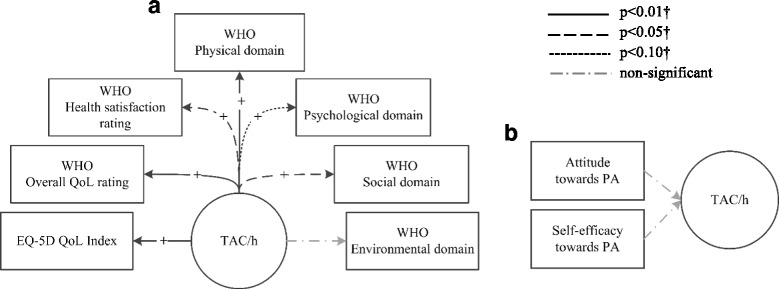
Summary of the relationships found, controlled for patient and disease characteristics^*^.(a) between physical activity and quality of life. (b) between attitude/self-efficacy and physical activity. TAC/h = average Total Activity Counts per hour; QoL = Quality of Life; EQ5D = EuroQol-5D; WHO = World Health Organisation. *corrected for any added value to the prediction by gender, age, diagnosis, years of hospitalization, illness severity and use of antipsychotics and antidepressants †For at least one of the quartiles of TAC/h.

This study clearly showed that (A) PA was positively associated with QoL and a significant predictor of overall, physical, psychological and social QoL, and (B) there was no association between attitude and self-efficacy and PA, contrary to what we expected. These findings are summarized in Fig. 2.

### Associations with QoL

The positive association with QoL needs to be discussed in more detail, as for overall, psychological and social QoL, relationships with PA were strongest for the patients classified in the second quartile. QoL of the most active patients was not significantly different from the QoL of the least active patients (reference group). As there were no big differences in daily programs during the week, weekend activities and approaching patients between wards, it is unlikely that this has affected results. Restlessness (caused by e.g. akathisia, which is well-known in this population [40], anxiety or agitated depression) could h`owever be a reason for high activity scores, while having a QoL below average. Nevertheless, adding presence of akathisia (which was available in the database and diagnosed by using the St. Hans Rating Scale [41]) to the models did not change the shape of the relations. Despite that an anxiety disorder was diagnosed only once and a diagnosis of agitated depression is also rare in this population, anxious and agitated feelings are common within psychiatric patients. Especially these feelings can be reflected in perceived psychological and social QoL, which may have been more common in patients with relatively high PA scores within the current study. However, it seems that relevant improvement in QoL can be achieved by activating the most sedentary patients to do/perform more light intensity PA. Intervention studies are needed to analyse such possible causal relationships. Noteworthy are the relatively low levels of explained variance of the models of the significant predictors (8% – 17%). This reflects the complexity of QoL, whereby – apart from patient and disease characteristics and PA – many more factors may play a role. In the QoL analysis, PA was not related to environmental QoL, in both association and prediction models. Years of hospitalization, illness severity and use of antipsychotics were the only significant predictors of environmental QoL. This could be a plausible finding for this population, since the hospitalized setting is a regulated environment itself, which is strongly related to one’s pathology and hardly affected by the individual level of physical activity.

These findings show only limited agreement with earlier cross-sectional research on the relationship between PA and QoL in inpatients with SMI. Two studies showed self-reported PA to be (positively) associated only with physical QoL [42, 43], while two others found no significant associations with any QoL variable [8, 18]. These differences may have to do with the power in these studies (*N* = 18 to 60) and the use of self-reports instead of objective measurement of PA.

It is important to perform research in specific SMI groups such as long-term hospitalized patients. Namely, the WHO QoL domain scores were on average 1.5 points higher compared to Dutch psychiatric outpatients and (just) 1.5 points lower than the general Dutch population (on a scale from 4 to 20) [44]. Because the QoL is not as low as we sometimes may assume in clinical practice and the non-linear relationships we found, the question is to what extend more PA can positively influence QoL in this hospitalized population. Current data suggests that slight increases in PA may be enough to improve QoL.

### Attitude/self-efficacy and physical activity

Results showed that SMI inpatients who have a positive attitude towards being physically active and feel able to do so, are not more active than SMI inpatients with a less positive attitude and lower self-efficacy. This might play a role in difficulties activating patients in clinical practice, taking into account that attempts to activate people often involve increasing attitude (e.g. telling them the importance and advantages of PA) and supporting self-efficacy (e.g. by facilitating them with home-trainers at the ward). A previous study showed similar results for self-efficacy, finding no association with exercise attendance in outpatients with SMI [45]. The idea that having a positive attitude and high self-efficacy towards PA not necessarily leads to increased PA, is described as the intention-behaviour gap [46]. In this population, a possible explanation for this gap could be a lack of competences to plan the actual activity, due to poor skills in cognitive abilities and systematic thinking [20, 21]. Also, based on clinical practice, other attitudes may play a role that compete with healthy behaviour (e.g. if a patient intended to take a walk but ends up with a fellow patient to smoke a cigarette). Nevertheless, although these factors may explain the lack of a relationship between attitude and self-efficacy and PA, this finding guides the way in which we should activate this population. Implementation intentions whereby a person plans when, where and how to act in an if-then format [47] are assumed to improve the translation of one’s intention to actual behaviour. However, due to the planning deficits [21], this could be less applicable in this population and a more intensive and guided approach might help to overcome this gap. Support by positive staff attitudes, staff participation (modelling) and peer support are determined as essential factors to affect ones’ behaviour to achieve a maximum result with a lifestyle intervention in patients with SMI [48]. Recent systematic reviews on this subject encourage structural physical activities integrated in a multidisciplinary treatment [6, 13, 49]. Moreover, provided that the obesogenic environment will be changed using such an activating strategy, especially inpatient healthcare offers a lot of opportunities. The inpatient setting has the abilities to structurally activate patients to engage in PA and pay attention to the development of habits they can maintain when discharged from the hospital [49].

### Strengths and limitations

So far, little is known about the level of PA of long-term hospitalized patients with SMI. The main strength of the present study is the use of objective measurement of PA in a relatively large group of long-term hospitalized patients with SMI [9] and involving modifiable psychological determinants. This leads to more reliable conclusions, independent of patient and disease characteristics. Accelerometer data are more reliable than self-reports due to psychopathology and impaired cognitive abilities of patients [50] and better registration of the lower spectrum of PA intensity [51]. Besides, by measuring PA in daily clinical practice and by involving modifiable psychological determinants such as attitude and self-efficacy, results are very relevant for clinical practice. Especially, research towards specific groups can contribute to better treatment, such as in long-term hospitalized population with SMI. Findings, such as the QoL scores compared to outpatients and non-linear distributions, show us that patients with SMI in different settings also differ in relationships between PA and QoL and therefore need a different approach.

The cross-sectional design of the present study does not allow the drawing of any conclusions about causality of the relationships. Another limitation is that there is no international consensus on how to set up and process accelerometer data. Besides, validated instruments to assess attitude and self-efficacy are not available in this population, because so far little research on this topic has been done in long-term hospitalized patients with SMI. The self-efficacy questionnaire was slightly adjusted and translated to Dutch by people with excellent command of English, but we did not perform an extensive back and forth translation-procedure. However, as shown in the method section, internal consistencies of the questionnaires were high. Moreover, the levels of explained variance of the models confirm the complex constructs of QoL, in which many individual aspects may play a role. Furthermore, in step-wise regression choices of elimination are based on the current data-set, which means that this procedure possibly reduced generalizability of the results. However, in our opinion this was the best way to achieve the maximum power of models to estimate and predict the relationships as accurately as possible, taking our sample-size into account.

We encourage further research on this topic within hospitalized SMI patients, especially regarding the non-linear relationship between TAC/h and QoL. For example, it would be very relevant for clinical practice to know whether there is a causal relationship between high levels of PA and lower QoL. If this is the case, it must be taken into account in future interventions aiming to improve the poor health status of this population by increasing PA. For such interventions, the lack of association between attitude and self-efficacy and PA suggests the need of a more intensive and guided approach. Therefore, we think that long-term hospitalized patients with SMI will benefit from an integrated, multidisciplinary and peer supported approach focusing on a shift from sedentary behaviour to light intensity PA.

## Conclusions

In summary, the current study showed a positive relationship between PA and QoL, especially for patients in the lower spectrum of PA. Although no causal relationship can be drawn, the data suggests that in long-term hospitalized patients with SMI slight increases in PA may be enough to improve QoL. The lack of association between attitude and self-efficacy and PA suggests the need of a more intensive and guided approach. We encourage further (longitudinal) research to obtain more accurate insights on this topic, which remains priority in this population.

## Abbreviations

EQ-5D: EuroQol five-dimension questionnaire
LOESS: Locally Estimated Scatterplot Smoothing
LPA: Light Physical Activity
MVPA: Moderate to Vigorous Physical Activity
PA: Physical Activity
Q: Quartile
QoL: Quality of Life
SB: Sedentary Behaviour
SMI: Severe Mental Illness
TAC/h: average Total Activity Counts per hour
WHOQoL-Bref: World Health Organization Quality of Life questionnaire, brief version.
